# Outcome of cardiopulmonary resuscitation in cancer patients in an Indian tertiary cancer center

**DOI:** 10.1186/cc14519

**Published:** 2015-03-16

**Authors:** SN Myatra, N Prabhu, JV Divatia

**Affiliations:** 1 Tata Memorial Hospital, Mumbai, India

## Introduction

Cardiopulmonary resuscitation (CPR) after cardiac arrest in cancer patients is often discouraged as it is associated with poor outcome. In our 700-bed tertiary cancer hospital in Mumbai, India, the ICU runs an in-hospital cardiac arrest team (CAT). We reviewed our data to determine outcome from CPR, identify factors associated with improved outcomes and justify the presence of a CAT in our cancer hospital.

## Methods

All in-hospital patients from November 2012 to November 2014 (2-year period) with unanticipated cardiorespiratory arrests were included. Data were recorded prospectively using the Utstein template. Only patients with cardiac arrest rhythms were included. Patients with anticipated progression towards arrest, those with seizures, hypotension without dysarrythmias or other medical emergencies were excluded. The outcomes studied were return of spontaneous circulation (ROSC) and survival on hospital discharge (SOHD). Binary logistic regression analysis was performed to determine factors associated with ROSC and SOHD.

## Results

One hundred and ninety-three patients (110 males, 83 females, mean age 48.2 ± 18.3 years) were studied. The mean time interval between collapse and onset of resuscitation was 2.3 ± 2.1 minutes. A total of 65.3% arrests were witnessed. Sustained ROSC occurred in 36.8% patients and the SOHD was 24.9%. The initial rhythm recorded during CPR was asystole in 133 patients, pulseless electrical activity in 21 patients and ventricular fibrillation/tachycardia (VF/VT) in 39 patients. SOHD for these rhythms was 8.3%, 33.3% and 76.9%, respectively. On univariate analysis, type of rhythm, witnessed arrests and time to resuscitation were associated with sustained ROSC and SOHD. On multivariate analysis, only type of rhythm, VF/VT (*P *= 0.000) and PEA (*P *= 0.017), were significantly associated with SOHD, while witnessed arrest and time to resuscitation were not.

## Conclusion

Sustained ROSC occurred in 36.8% patients and the SOHD was 24.9%. A reduced response time, witnessed arrest and type of rhythm are associated with ROSC and improved SOHD. The type of rhythm was independently associated with SOHD, with VF/VT and PEA having improved survival while asystolic patients had a poor outcome. These considerations justify the presence of a CAT in our cancer hospital.

